# Comparative Real-World Safety Profiles of Six Selective Serotonin Reuptake Inhibitors: A Global Pharmacovigilance Analysis

**DOI:** 10.7759/cureus.98677

**Published:** 2025-12-08

**Authors:** Adrian Chin Yan Chan

**Affiliations:** 1 Global Safety, Bayer Pharmaceuticals, Beijing, CHN

**Keywords:** adverse drug reactions, pharmacodynamics, pharmacovigilance, precision medicine, selective serotonin reuptake inhibitors

## Abstract

Background

Selective serotonin reuptake inhibitors (SSRIs) represent the cornerstone of modern antidepressant therapy, yet critical knowledge gaps persist regarding their comparative real-world safety profiles. This evidence deficit has profound implications for clinical decision-making and patient outcomes.

Methods

We conducted a comprehensive pharmacovigilance analysis utilizing VigiBase, the WHO global database of individual case safety reports, encompassing over 342,000 reports for six major SSRIs (sertraline, fluoxetine, paroxetine, citalopram, escitalopram, and fluvoxamine). Disproportionality analysis using information component (IC) values was performed across seven clinically relevant safety domains: anticholinergic effects, sexual dysfunction, metabolic effects, extrapyramidal symptoms, sleep disturbances, withdrawal syndrome, and cardiac conduction abnormalities.

Results

Significant heterogeneity in safety profiles was observed among SSRIs, with clear correlations between pharmacodynamic properties and adverse event patterns. Paroxetine demonstrated the highest rates of anticholinergic effects, sexual dysfunction, weight gain, and withdrawal syndrome, correlating with its high muscarinic M1 receptor binding affinity (Ki = 108 nM). Citalopram showed elevated cardiac conduction abnormalities, while fluoxetine exhibited increased extrapyramidal symptoms. A strong inverse relationship was observed between SSRI half-life and withdrawal syndrome reporting.

Conclusions

This analysis reveals that SSRIs exhibit distinct safety profiles that correlate with their pharmacodynamic properties, challenging the traditional view of these medications as a homogeneous therapeutic class. These findings support precision prescribing approaches based on individual patient risk factors and provide mechanistic insights for evidence-based SSRI selection.

## Introduction

Selective serotonin reuptake inhibitors (SSRIs) are among the most widely prescribed antidepressants, with more than 264 million prescriptions dispensed globally in 2023 [1]. They are effective for depression and anxiety disorders, yet important gaps remain in understanding their real-world safety profiles [2]. SSRIs increase synaptic serotonin by inhibiting the serotonin transporter (SERT) [3]. Although often considered similar, individual SSRIs differ in pharmacodynamic actions beyond SERT inhibition, including variable effects on noradrenergic, dopaminergic, histaminergic, and cholinergic systems [4-6]. These differences contribute to variations in adverse drug reactions (ADRs).

Most safety data originate from pre-marketing trials, which frequently exclude elderly patients, individuals with comorbidities, and those on multiple medications [7]. Trial conditions do not reflect real-world dosing patterns or long-term SSRI use [8]. Short study durations (typically 8-12 weeks) limit the detection of ADRs that appear with chronic therapy [9]. Regulatory systems such as the FDA Sentinel Initiative and EMA EudraVigilance attempt to improve post-marketing surveillance, but current real-world studies usually examine only specific drugs or single ADR categories [10-13].

Because SSRIs are prescribed for long periods for conditions such as major depressive disorder, generalized anxiety disorder, panic disorder, obsessive-compulsive disorder, and post-traumatic stress disorder, understanding their safety is essential [14]. ADRs cause 15-30% of treatment discontinuations, and the frequency differs among individual SSRIs [15]. With depression affecting over 280 million people worldwide, optimizing SSRI safety is a major public health need [16]. Recent pharmacoepidemiological studies also suggest differences in cardiovascular risk, arrhythmias, falls, fractures, and cognitive impairment across SSRIs, especially in older adults [17,18].

To address these gaps, we analyzed safety reports from VigiBase, the World Health Organization’s global adverse event reporting database [19]. VigiBase offers diverse population coverage, large sample sizes, real-world prescribing patterns, and long observational periods [20,21]. We examined six commonly used SSRIs, namely, sertraline, fluoxetine, paroxetine, citalopram, escitalopram, and another highly prescribed agent, to capture more than 90% of global SSRI exposure [22,23].

We focused on clinically relevant ADR domains frequently associated with long-term SSRI use. Anticholinergic effects vary substantially, with paroxetine showing the highest muscarinic receptor affinity, leading to cognitive impairment, dry mouth, constipation, and urinary retention, especially in older adults [24,25]. Sexual dysfunction is common, affecting 30-70% of users and often leading to discontinuation [26,27]. SSRIs can also cause weight changes that contribute to cardiovascular and metabolic risk [28]. Extrapyramidal symptoms such as akathisia and Parkinsonism occur through serotonin-dopamine interactions and may differ across agents [29,30]. Sleep disturbances, including rapid eye movement (REM) suppression and increased sleep latency, are also well-reported [31]. Withdrawal symptoms are frequent, especially with paroxetine and least with fluoxetine [32]. Finally, QT prolongation is a recognized risk, most strongly associated with citalopram [33,34].

Study objectives

The objectives of this study were as follows.

Primary objective: To characterize and compare the real-world safety profiles of six major SSRIs (sertraline, fluoxetine, paroxetine, citalopram, escitalopram, and fluvoxamine) using global pharmacovigilance data from VigiBase.

Secondary objectives: (a) To examine correlations between SSRI pharmacodynamic properties (receptor binding affinities) and adverse event reporting patterns across seven clinically relevant safety domains; (b) to explore relationships between pharmacokinetic parameters (elimination half-life) and withdrawal syndrome reporting; and (c) to provide mechanistic insights that can inform evidence-based SSRI selection and precision prescribing approaches.

## Materials and methods

Study data source

This study used data from VigiBase, the WHO global pharmacovigilance database, which contains over 19 million reports of suspected ADRs from more than 130 countries (representing >90% of the world’s population) [19]. These reports, submitted by member nations of the WHO Programme for International Drug Monitoring (PIDM) since 1968, include administrative, patient, medication, and ADR data sourced from healthcare professionals and patients. ADRs in VigiBase are coded using the Medical Dictionary for Regulatory Activities (MedDRA), a standardized five-level hierarchy (Table 1) that ensures consistent and reliable classification [35,36].

**Table 1:** The five-level hierarchy of MedDRA MedDRA: Medical Dictionary for Regulatory Activities

Level	Description	Example
1. System Organ Class (SOC)	Broadest category, based on anatomical or physiological systems.	"Gastrointestinal disorders"
2. High-Level Group Term (HLGT)	Subcategory within an SOC, grouping related conditions or symptoms.	"Gastrointestinal signs and symptoms"
3. High-Level Term (HLT)	Further subdivision of HLGT, focusing on specific types of conditions or symptoms.	"Nausea and vomiting symptoms"
4. Preferred Term (PT)	Specific adverse reaction or medical concept.	"Nausea"
5. Lowest-Level Term (LLT)	Most granular level, including synonyms, variants, or more specific descriptions.	"Nausea aggravated," "Nausea post-operative," "Feeling of nausea"

Data access and permissions

This analysis utilized VigiBase snapshot ER020-2024 (data lock point: June 4, 2024). All ADRs were coded using the MedDRA (version 27.0). Access to VigiBase data was obtained through a formal request to the WHO Collaborating Centre for International Drug Monitoring (Uppsala Monitoring Centre, UMC). The data were provided in accordance with the Guideline for Using VigiBase Data in Studies (version 4, March 2021) and the UMC Caveat Document (2021-11-10). The authors affirm that the information in this study comes from a variety of sources, and the probability that the suspected adverse effect is drug-related is not the same in all cases. The information presented does not represent the opinion of the UMC, the WHO, or any individual national pharmacovigilance centre.

Data extraction and deduplication process

A de-duplicated search was requested from VigiBase to minimize reporting bias. VigiBase employs vigiMatch, an algorithm using statistical modeling to identify suspected duplicate reports by scoring pairs of reports based on matching and mismatching information. All case reports up to the data lock point reporting any of the six SSRIs as suspect or interacting medication were retrieved, with no distinction made between primary and secondary suspect drugs; all suspect medications were treated equally in the analysis. The extraction query was performed by UMC using WHODrug Global coding for medicinal products and MedDRA v27.0 for adverse reaction coding. The search retrieved all individual case safety reports (ICSRs) in which any of the six SSRIs (sertraline, fluoxetine, fluvoxamine, paroxetine, citalopram, escitalopram) were recorded as suspect or interacting drugs. Reports were deduplicated using vigiMatch, a probabilistic algorithm that identifies potential duplicates by scoring pairs of reports on matching and mismatching parameters (drug name, reaction term, age, sex, country, and date of onset). The deduplicated dataset was supplied as an aggregated extract by UMC.

Access to VigiBase data was obtained through a formal request to the WHO VigiBase team, which typically grants access to research institutions and regulatory agencies for pharmacovigilance studies. As no patient identifiers were accessed and the analysis involved aggregated, retrospective data, ethical approval was not required for this research.

Design and definition

This was a retrospective observational study using disproportionality analysis to evaluate the safety profiles of six commonly prescribed SSRIs: sertraline, fluoxetine, fluvoxamine, paroxetine, citalopram, and escitalopram. All cumulative reports in VigiBase from the date of each drug’s first approval worldwide (i.e., its international birth date) up to 8 June 2024 were included.

The analysis focused on seven key safety topics, selected due to their clinical relevance, impact on long-term SSRI tolerability, and prominence in scientific literature. For each topic, disproportionality signals were assessed using MedDRA Preferred Terms most representative of the condition, ranging from formal diagnoses to hallmark symptoms. Of note, duplicate reports were removed by the VigiBase team to ensure the accuracy of disproportionality analyses.

Inclusion and exclusion criteria

Inclusion criteria comprised all reports in VigiBase containing any of the six SSRIs recorded as suspect or interacting drugs. Reports lacking a valid patient, reporter, or suspected drug, as defined under ICH E2B(R2) format, were excluded automatically by UMC’s data validation process. No further exclusions were applied based on seriousness, age, or region. Reports in all languages were included.

Data completeness and validity

Only reports meeting the minimum ICH E2B requirements (identifiable patient, identifiable reporter, adverse reaction, and suspected drug) were eligible. Each report’s completeness was assessed internally by UMC using VigiGrade scoring, which evaluates the amount of clinical and administrative information available.

Statistical analysis

Disproportionality analysis was conducted for all drug-event combinations within this dataset. The statistical measure of disproportionate reporting is the information component (IC) and is calculated from (1) the total number of reports in the database (Ntot), (2) the total number of reports on the ADR term (Nadr), (3) the number of reports on the drug (Ndrug), and (4) the total number of reports on the specific drug‐ADR combined (Ncomb).

The IC is calculated using Bayesian methodology to measure disproportionate reporting. The IC025 represents the lower limit of the 95% credibility interval for the IC value. A signal detection threshold of IC025 > 0 was applied, indicating that the observed reporting frequency is statistically higher than expected based on the background reporting in the database.

The IC is derived from (1) Ntot: total number of reports in VigiBase; (2) Nadr: total number of reports for each adverse event term; (3) Ndrug: total number of reports for each SSRI; and (4) Ncomb: number of reports for each specific SSRI-ADR combination.

No exclusions were made based on small counts; all IC results provided by VigiBase were included and handled equally in the analysis, regardless of the number of reports.

Reproducibility and data handling transparency

The IC and its 95% credibility interval (IC025) were calculated by UMC using the Bayesian Confidence Propagation Neural Network (BCPNN) model implemented in VigiBase. This approach quantifies disproportionate reporting based on the ratio of observed to expected drug-event combinations.

The IC value was not recalculated locally but obtained directly from the VigiBase output. All analyses in this manuscript were therefore performed using pre-computed IC and IC025 values from the VigiBase ER020-2024 dataset.

The IC does not imply causality; it quantifies the strength of association based on global reporting patterns.

Correlation analysis

Linear regression analysis was performed to examine the relationships between IC values and pharmacodynamic parameters (receptor binding affinities and half-life). For receptor binding affinity correlations, Ki values for the M1, 5-HT2C, and DAT receptors were log-transformed prior to analysis, as these measures exhibit a logarithmic distribution. The Ki value for the SERT receptor was analyzed without log transformation.

Correlation metrics, including Pearson correlation coefficients (r), p-values, and 95% confidence intervals, were obtained for all analyses and are displayed as labels adjacent to each respective regression line within the scatterplot diagrams. No corrections for multiple comparisons were applied. All statistical analyses were conducted using R (version 4.5.1; R Development Core Team, Vienna, Austria).

Regression and correlation parameterization

Correlation analyses were conducted using the lm() function in R, applying ordinary least squares regression without forced intercept terms. Pearson correlation coefficients (r) were computed using the cor.test() function with method="pearson". For each correlation, 95% confidence intervals and two-tailed p-values were obtained. No data transformations other than the specified log-transformation of Ki values were applied.

Receptor binding affinity data

All receptor binding affinity values (Ki, nmol/L) were obtained from Owens et al. [5], who employed radioligand binding assays measuring the concentration required for 50% inhibition of radioligand binding. The assays utilized human monoamine transporter preparations and receptor-specific radioligands. Detailed assay methodologies, including specific radioligands used, incubation conditions, and receptor preparation details, are available in the original publication.

Analysis of specific safety topics

Topic 1: Anticholinergic Side Effects

Central and peripheral anticholinergic adverse events were examined, focusing on disturbance in attention and mydriasis (dilated pupils). Scatterplots were generated to examine the relationship between IC values and the logarithm of SSRI binding affinities for the muscarinic cholinergic (M1) receptor. Additional central and peripheral anticholinergic adverse events were also examined.

Topic 2: Sexual Dysfunction

Sexual dysfunction events were analyzed, with particular focus on decreased libido. The relationship between IC values and SSRI binding affinities for the M1 receptor was examined through scatterplot analysis. Other adverse events pertinent to sexual dysfunction were also examined.

Topic 3: Weight Changes

Weight changes were evaluated through analysis of weight-increased and weight-decreased events. Scatterplots were generated to examine IC values against SSRI binding affinities for the M1 receptor (for weight increased) and the serotonergic 5HT-2C receptor (for weight decreased).

Topic 4: Extrapyramidal Reactions

Extrapyramidal reactions were assessed, focusing on Parkinsonism. The relationship between IC values and SSRI binding affinities was examined for multiple targets: the 5-HT reuptake transporter (SERT), the dopamine transporter (DAT), and the serotonergic 5-HT2C receptor. Additional extrapyramidal reaction events were also examined.

Topic 5: Sleep Disturbances

Sleep disturbances were analyzed through examination of insomnia and sleep disorder events. Additional sleep-related adverse events were also examined.

Topic 6: Withdrawal Effects

Due to substantial overlap between signs and symptoms of antidepressant discontinuation, other known SSRI adverse effects (e.g., amnesia, reduced concentration, nausea, vomiting), and primary disease symptoms (e.g., anxiety, suicide thoughts), analysis was restricted to events that clearly denote withdrawal (i.e., the MedDRA Preferred Term "Withdrawal syndrome"). The relationship between IC values and SSRI binding affinities for the muscarinic anticholinergic (M1) receptor and SERT was examined. Additionally, the correlation between SSRI half-lives and disproportionate reporting of withdrawal effects was explored.

Topic 7: QT Prolongation

QT prolongation events were examined, focusing on QT prolongation and torsade de pointes. Additional QT prolongation events were also analyzed.

Pharmacodynamic rationale for receptor selection

M1 muscarinic receptor for anticholinergic effects: Anticholinergic adverse events are primarily mediated through blockade of muscarinic acetylcholine receptors in the parasympathetic nervous system. The M1 receptor subtype was selected as the only muscarinic receptor subtype with comprehensive binding affinity data available in the Owens et al. [5] reference source, which provided the most complete comparative dataset across all six SSRIs examined.

5-HT2C receptor for weight changes: The serotonergic 5-HT2C receptor possesses activating properties that influence appetite regulation and energy metabolism. Activation of 5-HT2C receptors is associated with decreased food intake and weight loss, while antagonism promotes weight gain. This receptor was therefore selected to examine correlations with weight-decreasing events.

M1 receptor for sexual dysfunction and weight gain: In addition to classical anticholinergic effects, M1 receptor binding was examined in relation to sexual dysfunction and weight gain due to emerging evidence suggesting cholinergic pathways contribute to these adverse events beyond purely serotonergic mechanisms.

SERT for extrapyramidal symptoms and withdrawal: High SERT binding affinity and occupancy are fundamental to SSRI pharmacology. For extrapyramidal symptoms, excessive serotonergic activity in the basal ganglia may disrupt serotonin-dopamine balance. For withdrawal syndrome, high SERT affinity combined with short half-life creates rapid changes in transporter occupancy upon discontinuation.

DAT and 5-HT2C for extrapyramidal symptoms: DAT binding affinity was examined as dopaminergic activity directly influences motor control pathways. The 5-HT2C receptor was also evaluated given its role in modulating dopaminergic neurotransmission in the nigrostriatal pathway.

## Results

Table 2 presents the total number of ICSRs and unique drug-event combinations for each SSRI. Sertraline and fluoxetine had the highest ICSR counts, while fluvoxamine had the lowest. This trend aligns with global prescription patterns: fluoxetine (introduced in 1987) and sertraline (early 1990s) have been widely prescribed worldwide [30], whereas fluvoxamine's use remains indication-specific (primarily for obsessive compulsive disorder and social anxiety disorder) and less common [37]. The ICSR volume thus reflects estimated cumulative patient exposure, with more widely prescribed SSRIs naturally generating more reports.

**Table 2:** Total number of adverse events reported for each SSRI in the VigiBase dataset ICSR: individual case safety reports; SSRI: selective serotonin reuptake inhibitor

SSRI name	Number of ICSR reports	Number of unique drug-event combinations
Fluoxetine	82,760	4879
Citalopram	41,205	4170
Escitalopram	43,557	4501
Fluvoxamine	11,056	1739
Sertraline	88,298	5597
Paroxetine	75,625	4808

Topic 1: Anticholinergic side effects

**Figure 1:**
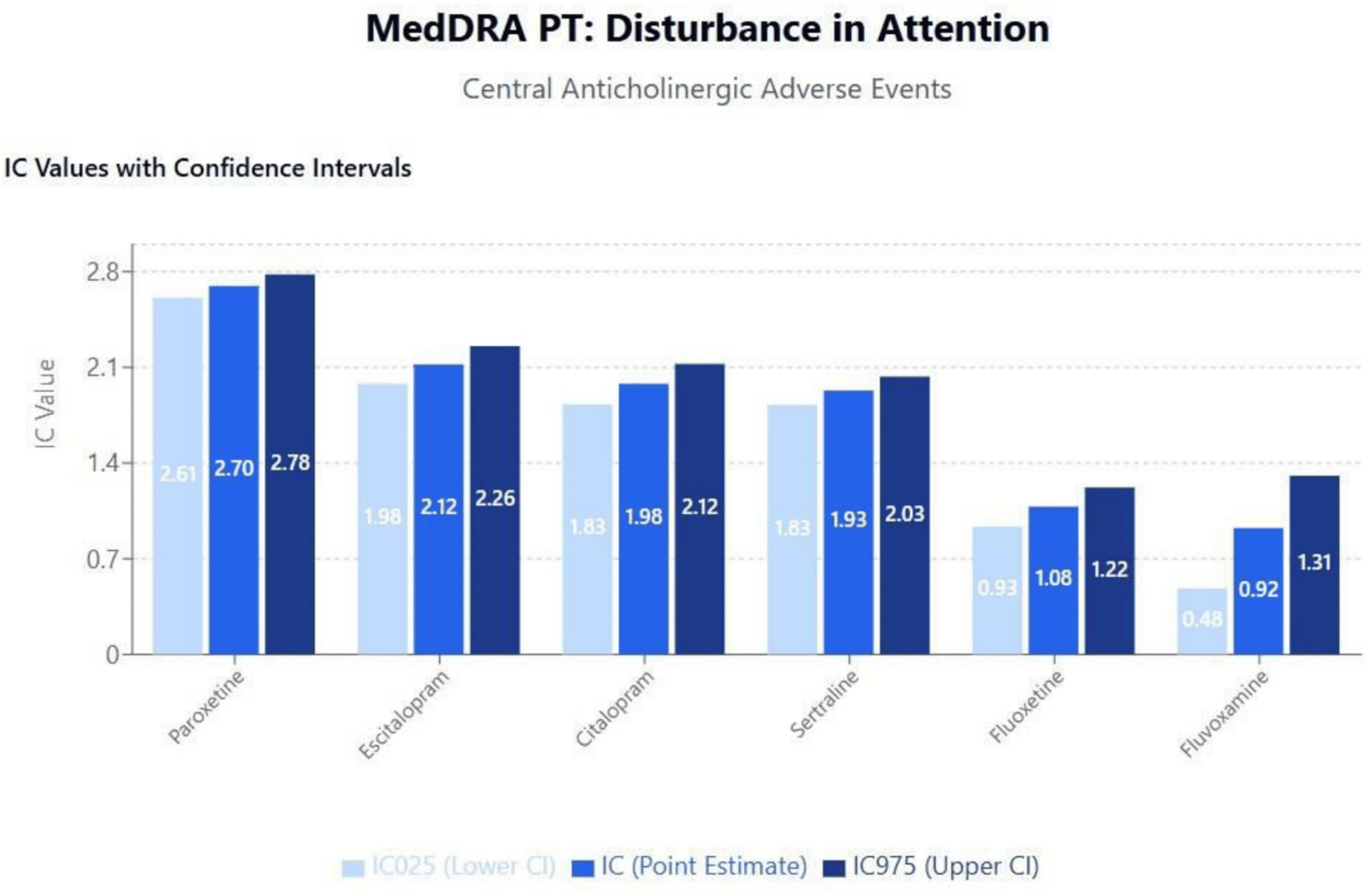
MedDRA PT: Disturbance in attention (central anticholinergic adverse events) MedDRA: Medical Dictionary for Regulatory Activities

**Figure 2:**
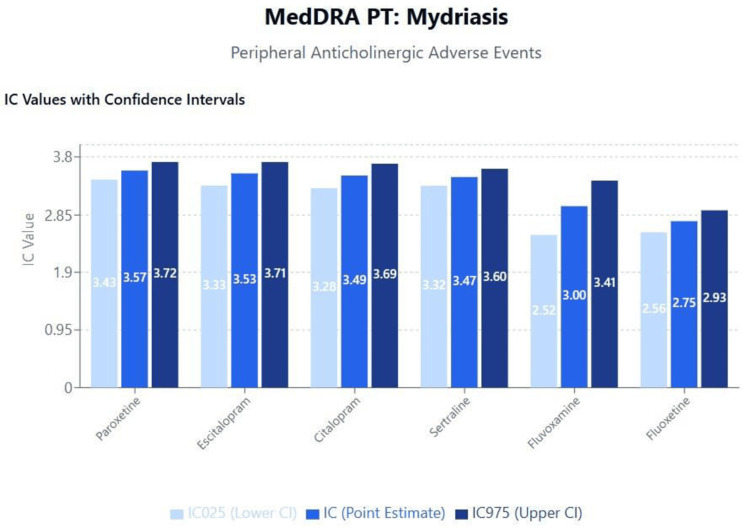
MedDRA PT: mydriasis (peripheral anticholinergic adverse events) MedDRA: Medical Dictionary for Regulatory Activities

**Figure 3:**
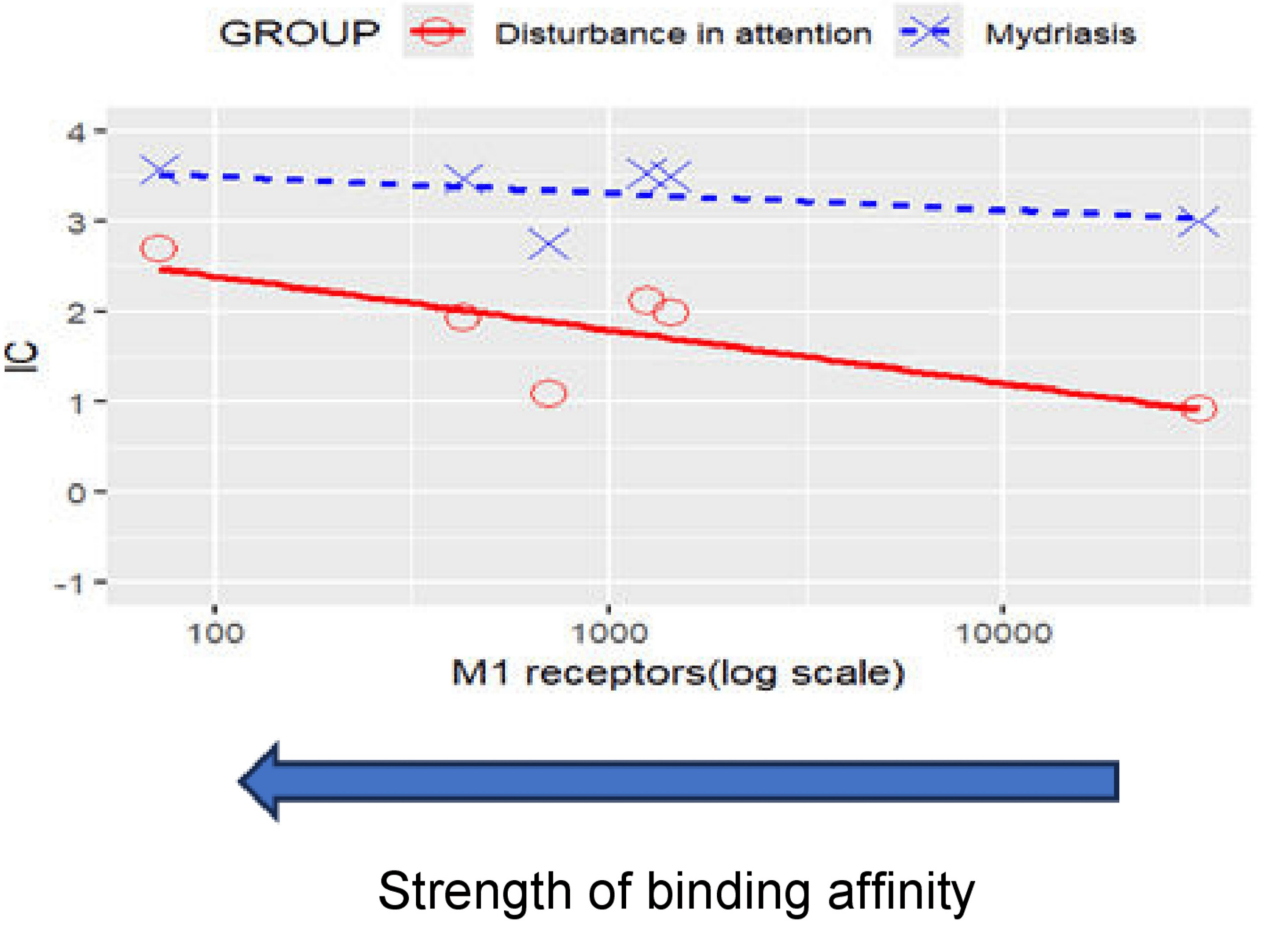
Scatterplot of IC values against SSRI binding affinity for the M1 receptor for anticholinergic events Disturbance in attention: Correlation between M1 receptors (log scale) and IC: r=-0.641, p=0.170>0.05; Mydriasis: Correlation between M1 receptors (log scale) and IC: r=-0.434, p=0.390>0.05. Note that a positive correlation can be observed between an SSRI’s binding affinity for the muscarinic M1 receptor and the disproportionate reporting of anticholinergic events. SSRI: Selective serotonin reuptake inhibitor

Results of disproportionality analyses for central and peripheral anticholinergic adverse events are shown in Figures 1-2 for disturbance in attention and mydriasis (dilated pupils), respectively. A scatterplot of IC values against the logarithm of SSRI binding affinities for the M1 receptor is shown in Figure 3. The results and trends observed with other anticholinergic events were generally consistent with those presented.

Topic 2: Sexual dysfunction

**Figure 4:**
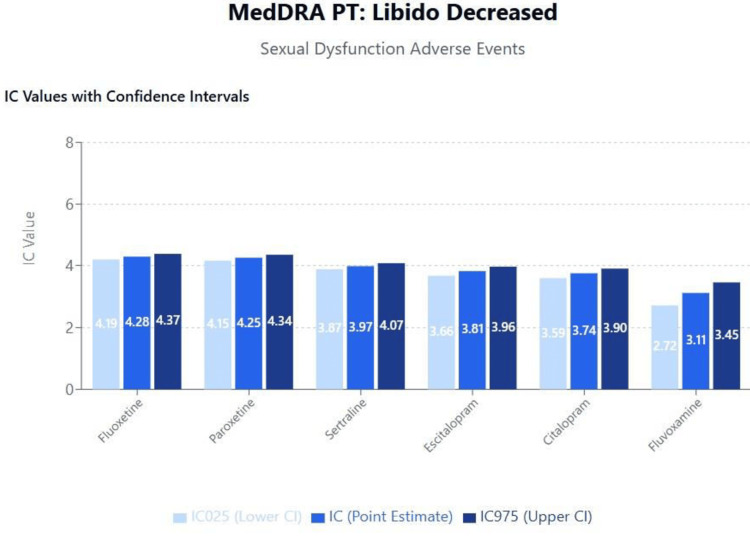
MedDRA PT: libido decreased (sexual dysfunction adverse events) MedDRA: Medical Dictionary for Regulatory Activities

**Figure 5:**
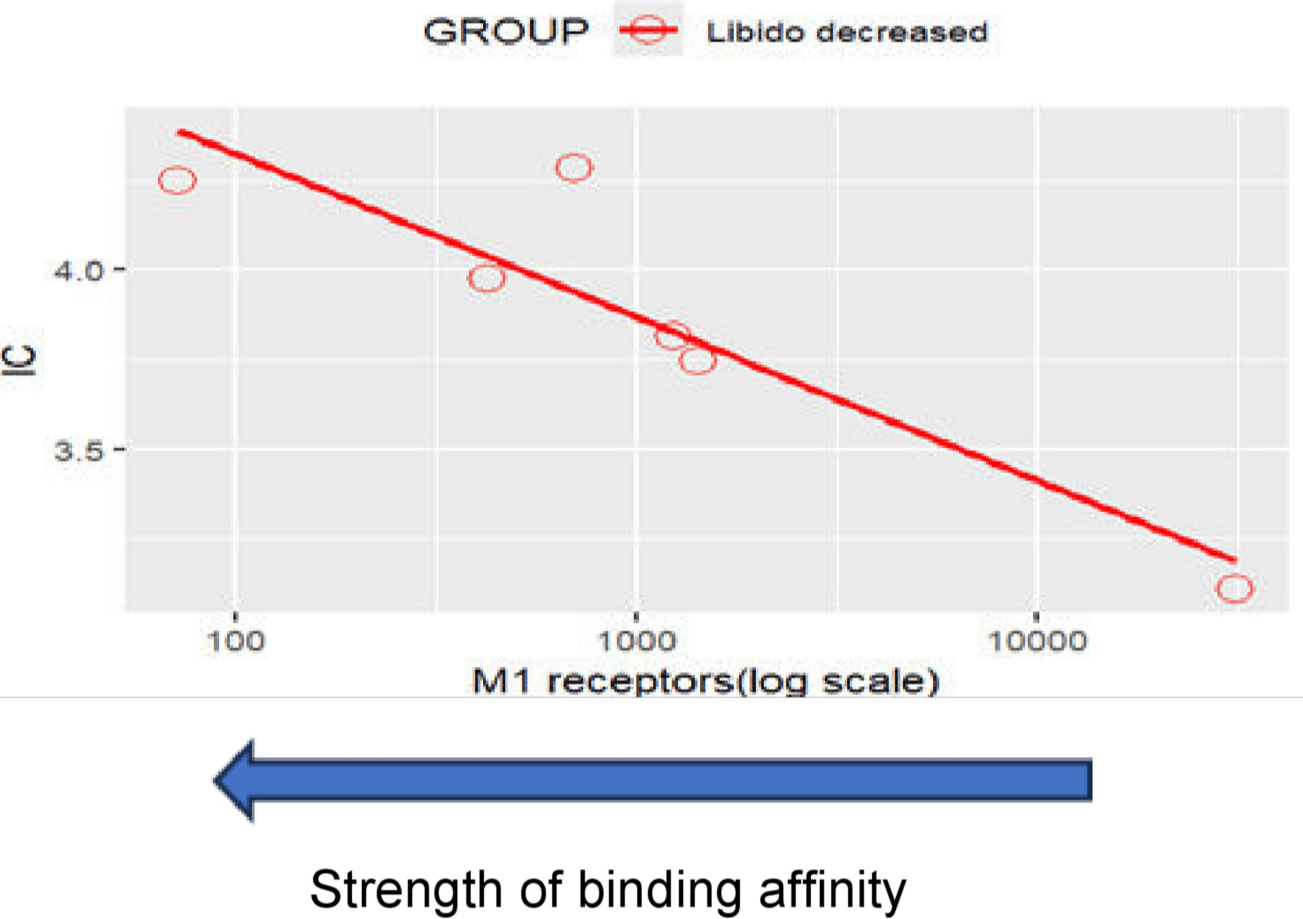
Scatterplot of IC values against SSRI binding affinity for the M1 receptor for sexual dysfunction Libido decreased: Correlation between M1 receptors (log scale) and IC: r=-0.875, p=0.022<0.05. Note that a positive correlation can be observed between an SSRI’s binding affinity for the muscarinic cholinergic M1 receptor and the disproportionate reporting of Libido decreased. SSRI: Selective serotonin reuptake inhibitor

Results of disproportionality analyses for libido decreased are shown in Figure 4. A scatterplot of IC values against the logarithm of SSRI binding affinities for the muscarinic cholinergic (M1) receptor is shown in Figure 5. The results and trends observed with other sexual dysfunction events were generally consistent with those presented. 

Topic 3: Weight changes

**Figure 6:**
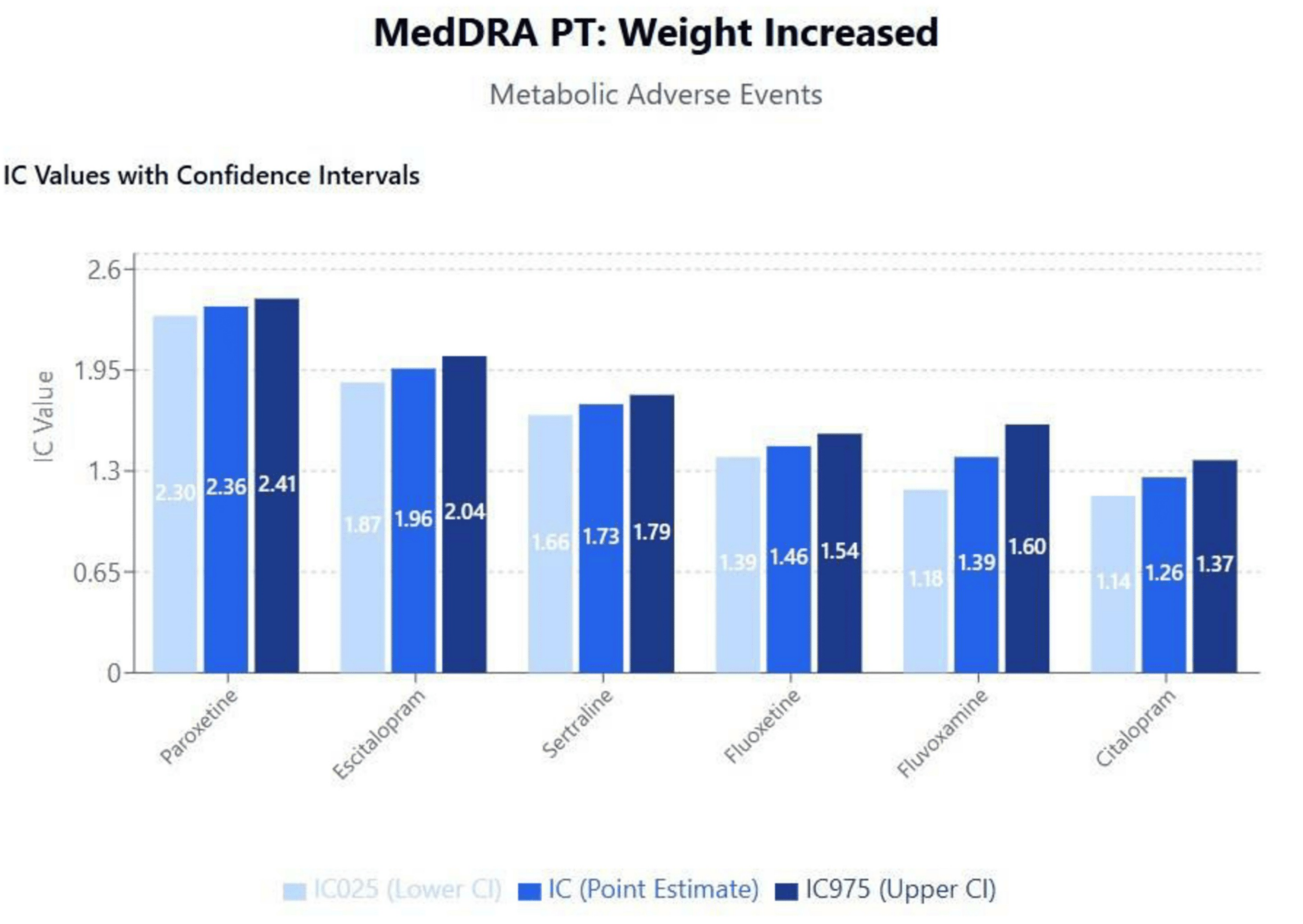
MedDRA PT: weight increased MedDRA: Medical Dictionary for Regulatory Activities

**Figure 7:**
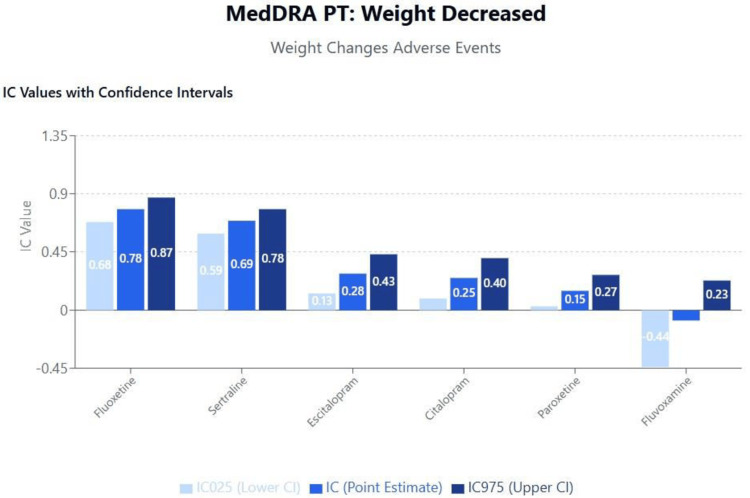
MedDRA PT: weight decreased (weight changes adverse events) MedDRA: Medical Dictionary for Regulatory Activities

**Figure 8:**
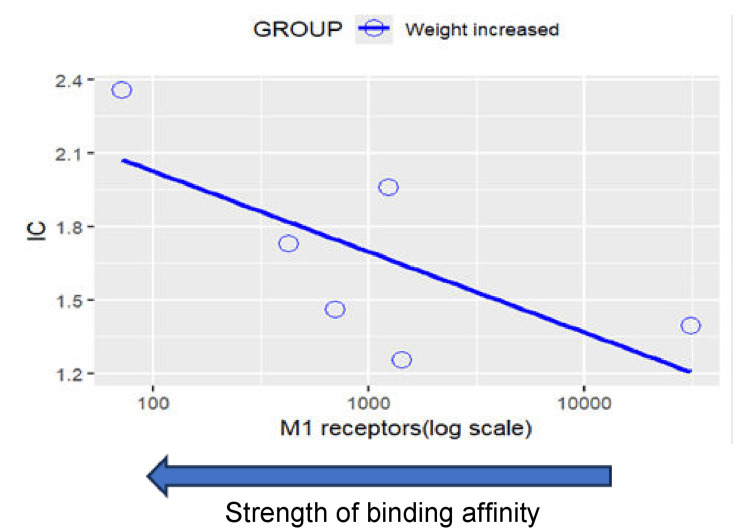
Scatterplot of IC values against SSRI binding affinity for the M1 receptor for the event weight increased Weight increased: Correlation between M1 receptors (log scale) and IC: r = -0.379, p = 0.458>0.05. Note that a positive correlation can be observed between an SSRI’s binding affinity for the M1 receptor and their disproportionate reporting of weight increased SSRI: Selective serotonin reuptake inhibitors

**Figure 9:**
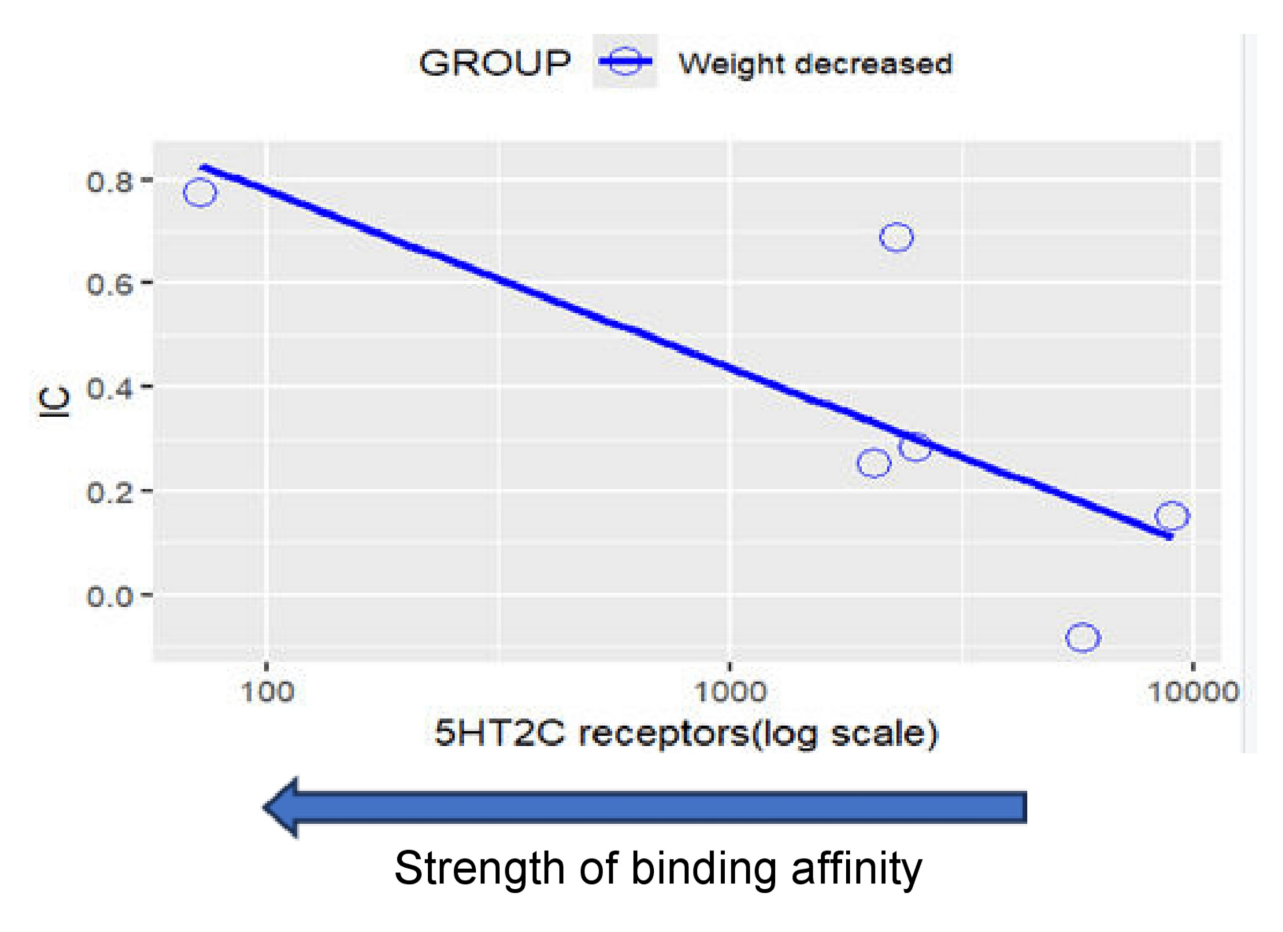
Scatterplot of IC value against SSRI binding affinity for the 5HT-2C receptor for the event weight decreased Weight decreased: Correlation between 5HT2C receptors (log scale) and IC: r = -0.710, p = 0.114>0.05. Note that a positive correlation can be observed between an SSRI’s binding affinity for the 5HT-2C receptor and their disproportionate reporting of weight decreased. SSRI: Selective serotonin reuptake inhibitor

Results of disproportionality analysis for weight increased and weight decreased are shown in Figures 6-7. A scatterplot of IC values for weight increased against the logarithm of SSRI binding affinities for the muscarinic cholinergic M1 receptor is shown in Figure 8. A second scatterplot of IC values for weight decreased against the logarithm of SSRI binding affinities for the serotonergic 5HT-2C receptor is shown in Figure 9.

Topic 4: Extrapyramidal reactions

**Figure 10:**
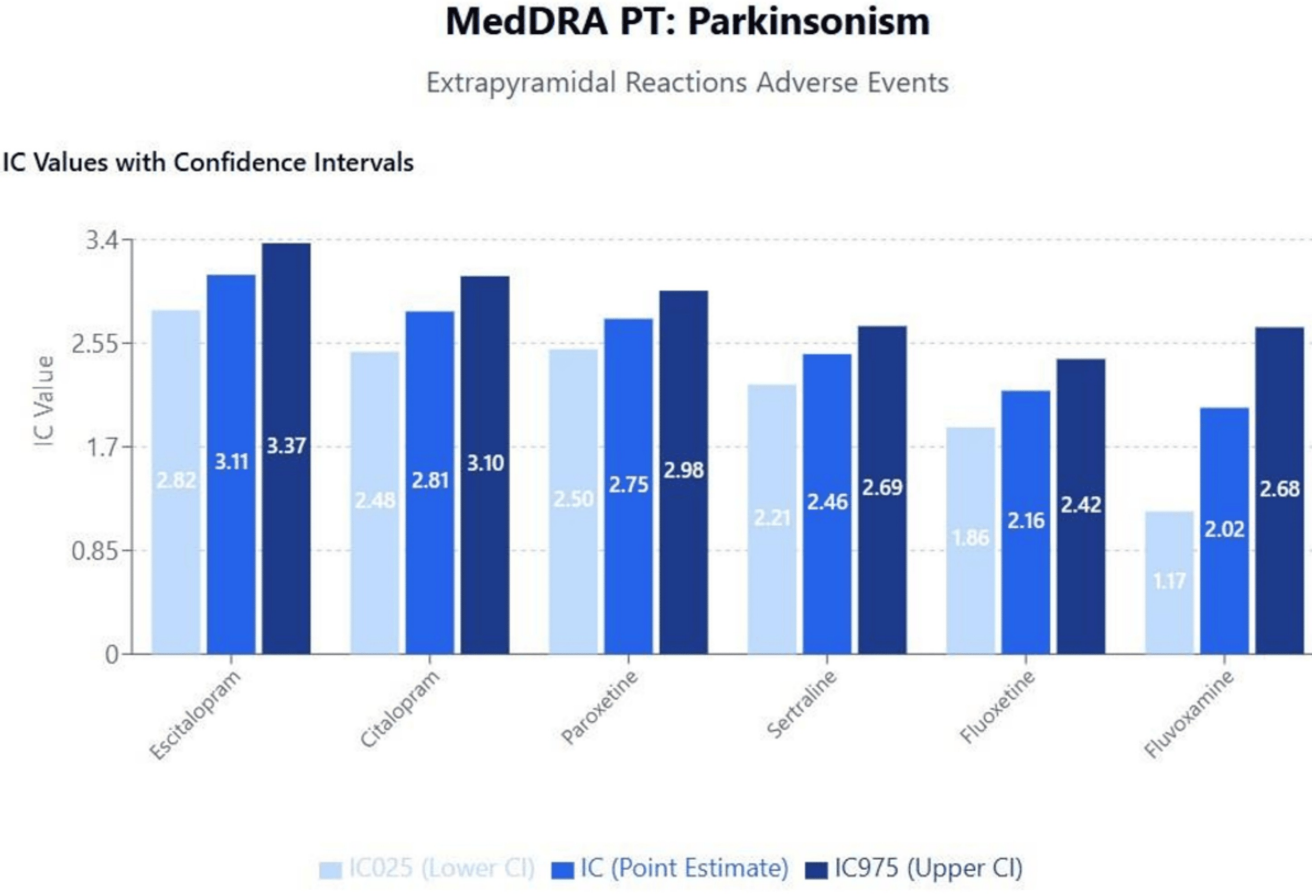
MedDRA PT: Parkinsonism (extrapyramidal reactions adverse events) MedDRA: Medical Dictionary for Regulatory Activities

**Figure 11:**
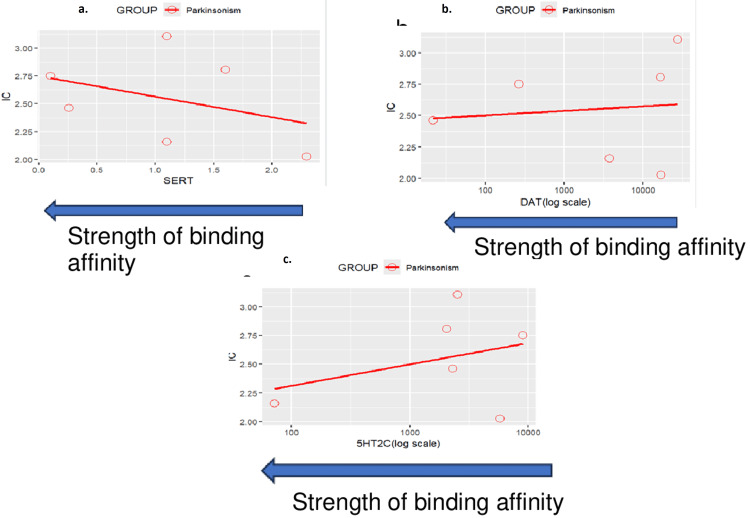
Scatterplot of IC values against various factors (a) Scatterplot of IC values against SSRI binding affinity for the SERT for the event of Parkinsonism (Note that a positive correlation can be observed between an SSRI’s binding affinity for the SERT and the disproportionate reporting of Parkinsonism). Parkinsonism: Correlation between SERT and IC: r = -0.368, p = 0.473>0.05. (b) Scatterplot of IC value against SSRI binding affinity for the DAT for the event of Parkinsonism (Note that a negative correlation can be observed between an SSRI’s binding affinity for the dopamine transporter and the disproportionate reporting of Parkinsonism). Parkinsonism: Correlation between DAT (log scale) and IC: r = 0.400, p = 0.432>0.05. (c) Scatterplot of IC value against SSRI binding affinity for the 5-HT2C for the event of Parkinsonism (Note that a negative correlation can be observed between an SSRI’s binding affinity for the 5-HT2C receptor and the disproportionate reporting of Parkinsonism). Parkinsonism: Correlation between 5HT2C (log scale) and IC: r = 0.067, p = 0.899>0.05. IC: information component; SERT: Serotonin transporter; SSRI: Selective serotonin reuptake inhibitor

Results of disproportionality analyses for Parkinsonism are shown in Figure 10. A scatterplot of IC values for Parkinsonism against the SSRI binding affinities for the 5-HT reuptake transporter (SERT) is shown in Figure 11a. A second scatterplot of IC values for Parkinsonism against the logarithm of SSRI binding affinities for the DAT is shown in Figure 11b. A third scatterplot of IC values for Parkinsonism against the logarithm of SSRI binding affinities for the serotonergic 5-HT2C receptor is shown in Figure 11c. The results and trends observed with other extrapyramidal reaction events were generally consistent with those presented.

Topic 5: Sleep disturbances

**Figure 12:**
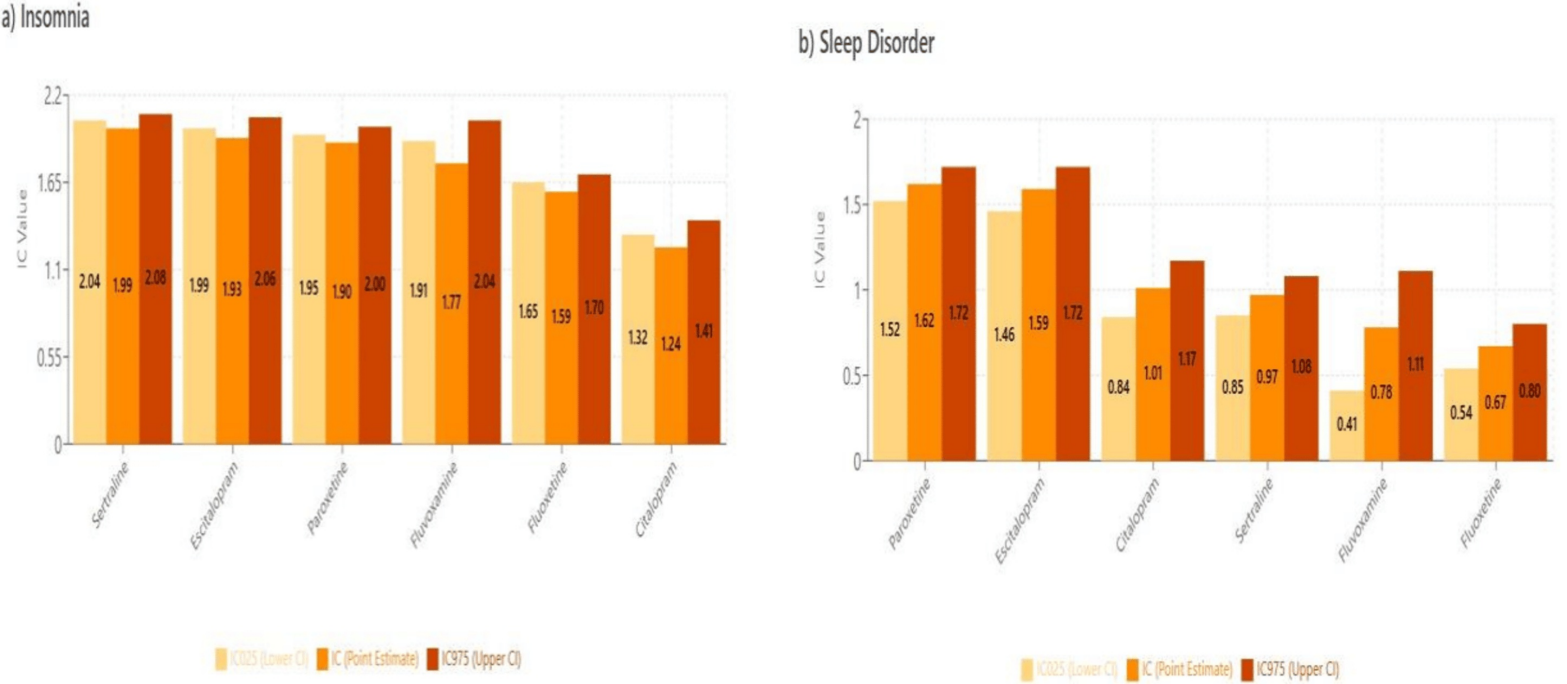
MedDRA PT: sleep disturbances MedDRA: Medical Dictionary for Regulatory Activities (a) Insomnia and (b) sleep disorder adverse events

Results of disproportionality analyses for insomnia and sleep disorders are shown in Figure 12.

Topic 6: Withdrawal effects

**Figure 13:**
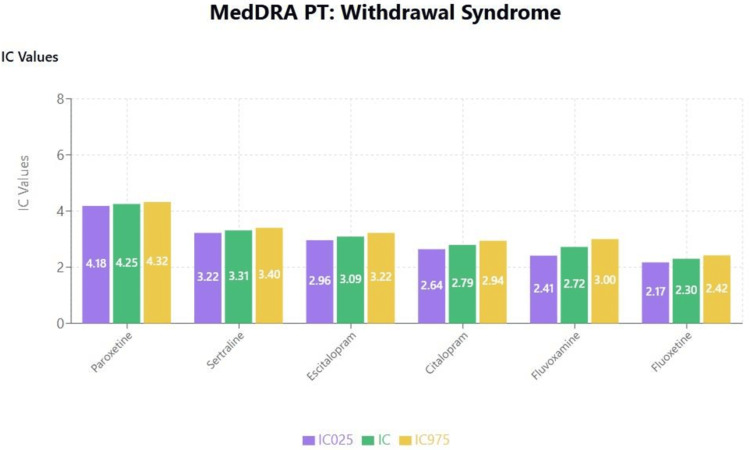
MedDRA PT: withdrawal syndrome MedDRA: Medical Dictionary for Regulatory Activities

**Figure 14:**
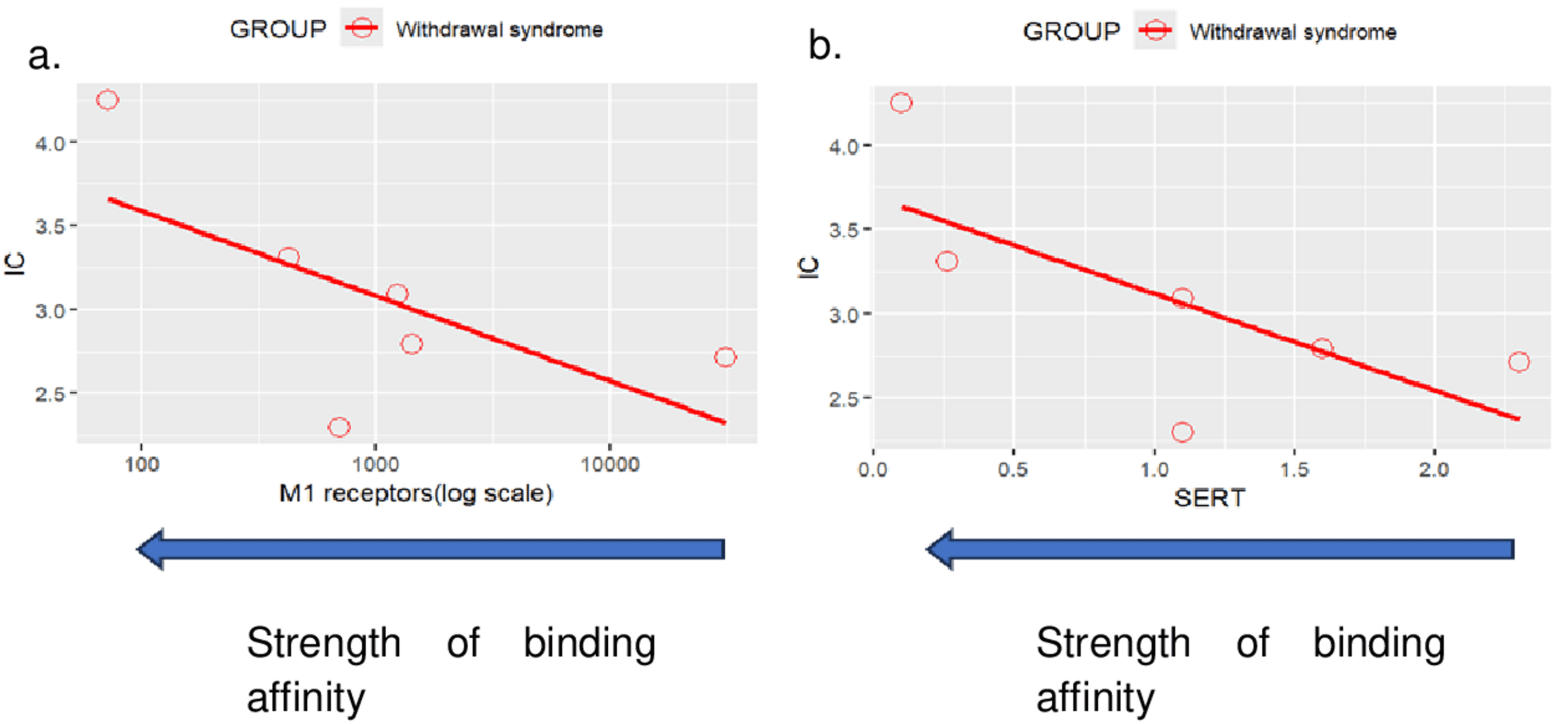
Scatterplot of IC values against various factors (a) Scatterplot of IC values against SSRI binding affinity for the M1 receptor for withdrawal syndrome (Note that a positive correlation can be observed between an SSRI’s binding affinity for the M1 receptor and the disproportionate reporting of Withdrawal syndrome). Withdrawal syndrome: Correlation between M1 receptors (log scale) and  IC: r = -0.286, p = 0.582>0.05. (b) Scatterplot of IC values against SSRI binding affinity for the SERT for withdrawal syndrome (Note that a positive correlation can be observed between an SSRI’s binding affinity for the SERT and the disproportionate reporting of Withdrawal syndrome). Withdrawal syndrome: Correlation between SERT and  IC: r = -0.703, p = 0.119>0.05.

Results of disproportionality analyses for withdrawal syndrome are shown in Figure 13. A scatterplot of IC values for withdrawal syndrome against the logarithm of SSRI binding affinities for the muscarinic anticholinergic (M1) receptor is shown in Figure 14a. A second scatterplot of IC values for withdrawal syndrome against the SSRI binding affinities for the SERT is shown in Figure 14b.

**Figure 15:**
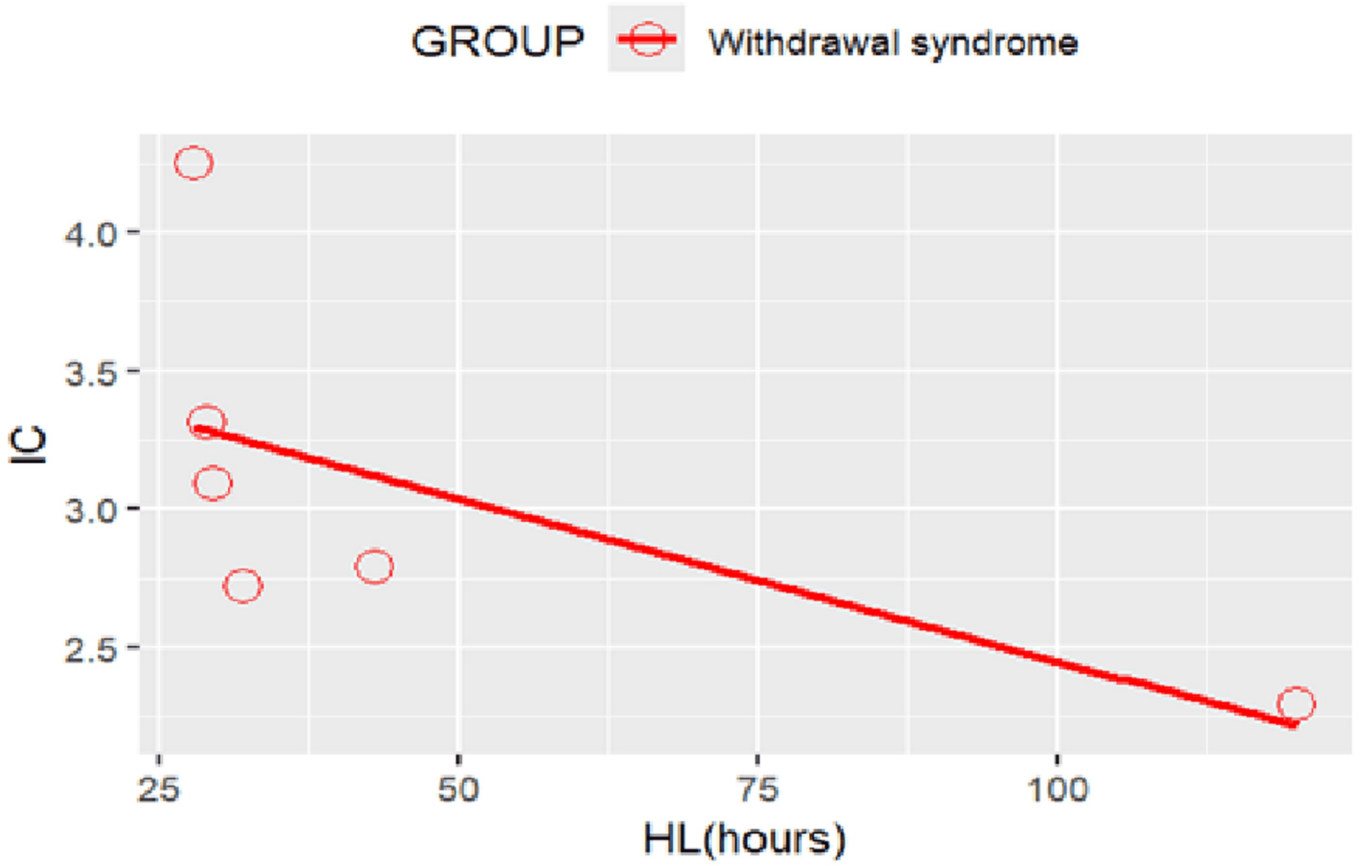
Scatterplot of IC values against SSRI half-life for event of withdrawal syndrome Withdrawal syndrome: Correlation between Half life and  IC: r = -0.635, p = 0.175>0.05. (Note that an inverse correlation can be observed between an SSRI’s half-life and the disproportionate reporting of withdrawal syndrome. IC: Information component; SSRI: Selective serotonin reuptake inhibitor

The half-lives of SSRIs as obtained from the literature are shown in Table 3. To explore the correlation between SSRI half-life and disproportionate reporting of withdrawal effects, a scatterplot of IC values for withdrawal syndrome against the half-life of each SSRI is shown in Figure 15.

**Table 3:** Half-lives of the six SSRIs in our study SSRI: Selective serotonin reuptake inhibitor

SSRI	Half-Life
Citalopram	38-48 hours
Escitalopram	27-32 hours
Fluoxetine plus N-methyl fluoxetine	4-6 days, 4-16 days
Fluvoxamine	21-43 hours
Paroxetine	12-44 hours
Sertraline	22-36 hours

Topic 7: QT prolongation

**Figure 16:**
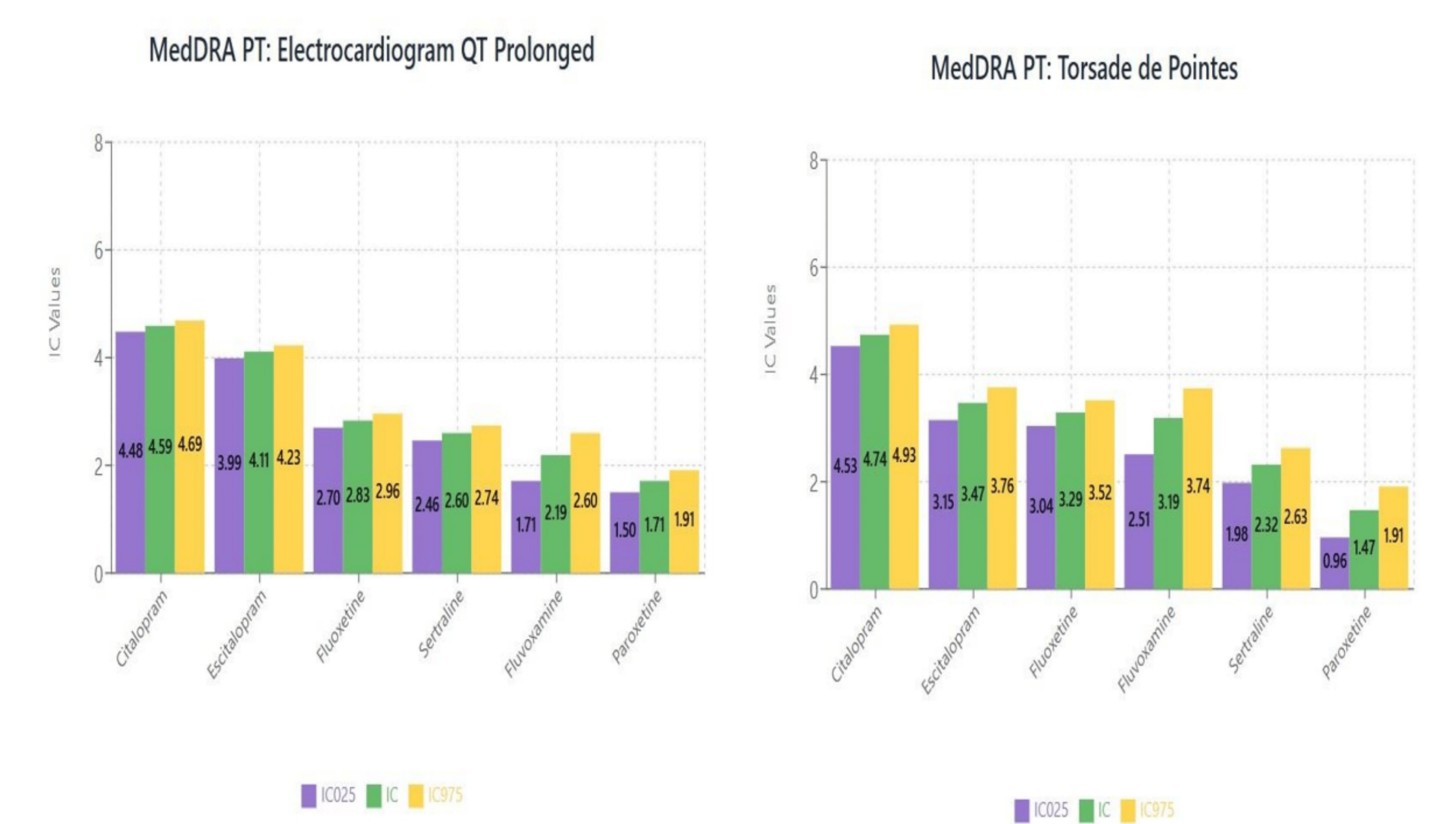
(a) MedDRA PT: electrocardiogram QT prolonged and (b) MedDRA PT: torsade de pointes MedDRA: Medical Dictionary for Regulatory Activities

Results of disproportionality analysis for QT prolongation and torsade de points are shown in Figures 16a-16b, respectively.

## Discussion

This comprehensive pharmacovigilance analysis of six major SSRIs using the WHO VigiBase database represents the largest comparative real-world safety evaluation to date, encompassing over 342,000 individual case safety reports across diverse global populations. Our findings demonstrate significant heterogeneity in adverse event reporting patterns among SSRIs, challenging the traditional view of these medications as a homogeneous therapeutic class. The observed patterns of adverse events demonstrate clear correlations with established pharmacodynamic properties, providing mechanistic insights that can inform evidence-based prescribing decisions.

The most striking finding was the pronounced association between muscarinic M1 receptor binding affinity and multiple adverse event categories, including anticholinergic effects, sexual dysfunction, weight gain, and withdrawal syndrome. This relationship was most evident with paroxetine, which demonstrated the highest binding affinity for the M1 receptor (Ki = 108 nM) and correspondingly elevated disproportionate reporting across these safety domains. These findings align with recent mechanistic studies demonstrating that anticholinergic activity significantly contributes to SSRI tolerability issues beyond the primary serotonergic mechanism [38,39].

Anticholinergic effects and cognitive implications

The clear correlation between M1 receptor binding affinity and central anticholinergic effects, particularly disturbance in attention, has significant clinical implications given the growing recognition of anticholinergic burden as a modifiable risk factor for cognitive decline and dementia [40,41]. Our findings suggest that paroxetine's high M1 receptor affinity may contribute to its association with cognitive impairment, particularly in elderly populations where anticholinergic medications are increasingly recognized as inappropriate prescribing choices [42].

Recent prospective cohort studies have corroborated these concerns, with Gray et al. [43] demonstrating that medications with strong anticholinergic properties, including paroxetine, increase dementia risk by 54% when used for more than three years. Similarly, a large-scale population study by Coupland et al. [44] found that anticholinergic antidepressants were associated with a 29% increased risk of dementia diagnosis. These findings support our pharmacovigilance signals and suggest that SSRIs with lower anticholinergic activity may be preferable for long-term treatment, particularly in cognitively vulnerable populations.

Sexual dysfunction

The differential reporting patterns for sexual dysfunction across SSRIs revealed unexpected correlations with anticholinergic activity rather than purely serotonergic mechanisms. This finding challenges the conventional understanding that SSRI-induced sexual dysfunction results exclusively from serotonin-mediated inhibition of sexual response pathways [45]. Recent neuroimaging studies have demonstrated that sexual function involves complex interactions between serotonergic, dopaminergic, and cholinergic systems, with anticholinergic medications potentially exacerbating sexual dysfunction through peripheral autonomic effects [46,47].

Our findings align with clinical observations suggesting that switching from high-anticholinergic SSRIs such as paroxetine to agents with minimal anticholinergic activity may improve sexual function outcomes. This mechanistic insight has practical implications for treatment selection, particularly given that sexual dysfunction affects 40-65% of SSRI users and represents a leading cause of treatment discontinuation [26,48].

Metabolic effects and long-term health outcomes

The relationship between SSRI use and weight changes revealed distinct patterns consistent with known pharmacodynamic properties. The positive correlation between M1 receptor binding and weight gain, most pronounced with paroxetine, aligns with clinical evidence demonstrating that anticholinergic medications promote weight gain through effects on appetite regulation and glucose metabolism [49,50]. Conversely, the association between 5-HT2C receptor binding and weight loss provides mechanistic support for the weight-neutral or weight-reducing effects observed with fluoxetine and sertraline.

These metabolic effects have significant long-term health implications, particularly given the association between SSRI-induced weight gain and increased cardiovascular risk, diabetes development, and treatment discontinuation [51,52]. Recent meta-analyses have confirmed that paroxetine is associated with significantly greater weight gain compared to other SSRIs, with mean differences of 2-4 kg observed in long-term studies [53,54]. These findings support the clinical practice of preferentially selecting SSRIs with favorable metabolic profiles for patients with obesity, diabetes, or cardiovascular risk factors.

Extrapyramidal reactions 

The complex relationship between SSRI pharmacodynamics and extrapyramidal reactions revealed in our analysis provides important insights into serotonin-dopamine interactions in movement control. The positive correlation between SERT binding affinity and Parkinsonism, coupled with negative correlations for dopamine transporter and 5-HT2C receptor binding, suggests that SSRIs with high serotonergic selectivity may paradoxically increase extrapyramidal symptom (EPS) risk through disruption of serotonin-dopamine balance in the basal ganglia [55,56].

While earlier literature and case reports have identified EPS with SSRIs such as fluoxetine and paroxetine [56], our VigiBase analysis revealed a relatively lower IC value for fluoxetine, suggesting a weaker signal for Parkinsonism compared to other SSRIs. This discrepancy may be accounted for by pharmacodynamic theories proposed by Guo et al. [30], who demonstrated that fluoxetine antagonizes 5-HT2 receptors on GABAergic interneurons, leading to disinhibition of dopaminergic neurons in the nigrostriatal pathway, thereby reducing the likelihood of EPS.

This explanation provides a plausible neurobiological mechanism that aligns with our real-world safety data, indicating that fluoxetine may pose a lower EPS risk than previously assumed. The apparent inconsistency between literature-based assumptions and pharmacovigilance findings underscores the importance of integrating receptor-level pharmacodynamics into safety signal interpretation.

Overall, our findings emphasize that EPS risk is not uniform across SSRIs and is likely influenced by complex serotonergic and dopaminergic receptor interactions, rather than solely by serotonergic potency. Further controlled studies are needed to validate these mechanistic interpretations.

Sleep architecture and circadian disruption

The differential effects of SSRIs on sleep disturbances observed in our analysis reflect the complex relationship between serotonergic neurotransmission and sleep-wake regulation. All SSRIs demonstrated significant associations with sleep disorders, consistent with their established effects on REM sleep suppression and sleep architecture disruption [57,58].

Fluoxetine, in particular, has been associated with more pronounced REM suppression and alerting effects due to its long half-life and activating profile, which may contribute to increased reports of insomnia in susceptible patients [57]. However, our analysis did not reveal stronger signals of disproportionate reporting for fluoxetine compared to other SSRIs regarding sleep disturbances. Further studies are needed to determine whether fluoxetine’s unique properties, as described above, may actually lead to more severe disruptions in sleep quality than other SSRIs or whether other yet discovered pharmacological mechanisms may also contribute to the differential effects of SSRIs on sleep quality observed in our analysis.

Furthermore, recent polysomnographic studies have demonstrated that SSRI-induced sleep disturbances can persist for months after treatment initiation and may contribute to treatment resistance in some patients [57]. The relationship between sleep disruption and antidepressant efficacy has gained increasing attention, with evidence suggesting that medications with less disruptive sleep profiles may achieve better long-term outcomes [58,59]. This highlights the importance of further research needed to better understand the mechanisms for SSRI-associated sleep disturbances and the need to minimize sleep disturbance as a side-effect while patients are on SSRI treatments.

Withdrawal syndrome: pharmacokinetic determinants

The clear inverse relationship between SSRI half-life and withdrawal syndrome reporting provides strong support for pharmacokinetic determinants of discontinuation symptoms. Paroxetine's combination of short half-life (12-44 hours) and high SERT binding affinity resulted in the highest withdrawal risk, while fluoxetine's extended half-life (4-16 days including active metabolites) conferred protective effects against discontinuation symptoms [60,61].

These findings have important clinical implications given that withdrawal symptoms affect 20-78% of patients discontinuing SSRIs and can significantly impact treatment compliance and quality of life [62,63]. Recent guidelines have emphasized the importance of gradual tapering protocols, particularly for short half-life SSRIs, with some experts recommending hyperbolic tapering schedules to minimize withdrawal risk [63,64].

Cardiac safety: QT prolongation and regulatory implications

The differential cardiac safety profiles observed in our analysis align with established knowledge regarding SSRI effects on cardiac conduction. Citalopram's elevated association with QT prolongation and torsade de pointes is consistent with FDA warnings issued in 2011 regarding dose-dependent QT prolongation risk [65]. Subsequent studies have confirmed that citalopram carries the highest cardiac risk among SSRIs, with significant QT prolongation observed at therapeutic doses [66].

The cardiac safety implications extend beyond QT prolongation, with recent large-scale cohort studies demonstrating increased risks of cardiovascular events, including myocardial infarction and stroke, associated with certain SSRIs [67,68]. These findings support the need for cardiac risk assessment before initiating SSRI therapy, particularly in elderly patients or those with pre-existing cardiovascular disease.

Clinical implications and treatment selection

The heterogeneous safety profiles revealed in our analysis support a more nuanced approach to SSRI selection based on individual patient characteristics and risk factors. Rather than treating SSRIs as interchangeable agents, clinicians should consider specific safety profiles when making treatment decisions. For example, patients with cognitive concerns may benefit from SSRIs with lower anticholinergic activity (sertraline, escitalopram), while those with metabolic risk factors might be better served by agents with weight-neutral profiles (fluoxetine, sertraline).

The concept of "precision prescribing" in psychiatry has gained increasing attention, with growing evidence that pharmacogenomic testing and individual risk profiling can improve treatment outcomes [69,70]. Our findings provide additional support for this approach by demonstrating that SSRI safety profiles can be predicted from pharmacodynamic properties, enabling more informed treatment selection.

Strengths of the study

This study has several methodological and analytical strengths. First, it utilizes VigiBase, the world’s largest and most diverse pharmacovigilance database, enabling broad global representation across healthcare systems and populations. Second, the study compares six widely used SSRIs within a single analytical framework (Appendix Figure 17 for the VigiBase data flow and analytical framework and Appendix Table 4 for the summary of data processing steps in the VigiBase-based analysis), providing a comprehensive and clinically meaningful comparison not previously reported. Third, the integration of pharmacodynamic and pharmacokinetic correlations with disproportionality signals offers mechanistic insights that extend beyond descriptive pharmacovigilance data. Finally, the use of pre-deduplicated, quality-controlled data from the UMC enhances the reliability of the findings.

Limitations 

Several limitations must be acknowledged in interpreting these findings, as they substantially constrain causal inference and generalizability.

First, pharmacovigilance databases are subject to multiple forms of reporting bias. Spontaneous reporting systems such as VigiBase capture only a fraction of actual adverse events, with reporting rates varying substantially by event type (serious events are over-reported relative to minor events), healthcare system (countries with mandatory reporting show different patterns than voluntary systems), and medication familiarity (newer drugs often receive heightened surveillance). Systematic reviews suggest the overall underreporting rates of 90-95% for adverse drug reactions, though this varies considerably across event categories [71,72]. Regional differences in pharmacovigilance culture and infrastructure mean that reporting patterns from high-income countries with established surveillance systems may dominate the database, potentially limiting generalizability to other settings.

Second, disproportionality analysis cannot establish causality. The observed associations between SSRIs and adverse events may be influenced by multiple unmeasured confounders. Specifically, our analysis could not adjust for (1) dosage and treatment duration critical factors affecting both efficacy and safety; (2) patient comorbidities which may independently contribute to adverse events and influence SSRI selection; (3) concurrent medications particularly relevant given high polypharmacy rates in psychiatric populations; and (4) indication for prescribing certain SSRIs may be preferentially prescribed for conditions associated with specific adverse events (confounding by indication). For example, paroxetine's preferential use in anxiety disorders, which are themselves associated with sleep disturbances and sexual dysfunction, may partially explain elevated reporting of these events.

Third, the correlation analyses examining relationships between pharmacodynamic parameters and safety signals are limited by a small sample size (n = 6 SSRIs). With only six data points, correlation coefficients have wide confidence intervals and limited statistical power to detect relationships, particularly non-linear associations. The absence of statistical significance (p > 0.05) for several observed trends should not be interpreted as evidence of no relationship, but rather as insufficient power to detect associations of the observed magnitude. Additionally, receptor binding affinity data were obtained from in vitro studies using standardized assay conditions that may not fully reflect in vivo pharmacology or account for active metabolites.

Fourth, the absence of exposure denominators prevents the calculation of absolute adverse event rates. Disproportionality measures (IC values) indicate whether an event is reported more frequently than expected for a given drug relative to the entire database, but cannot estimate true incidence or prevalence. Drugs with larger prescription volumes naturally generate more reports, and we cannot determine whether observed differences reflect true risk differences or differential exposure patterns.

Fifth, the analysis was limited to seven pre-specified safety domains selected based on clinical relevance and literature precedent. Other important adverse events (e.g., gastrointestinal effects, bleeding risk, hyponatremia) were not systematically examined and may reveal additional safety differences among SSRIs. The focus on specific MedDRA Preferred Terms may have missed relevant events coded under alternative terms.

Despite these limitations, the large sample size, global diversity, and long-term post-marketing observation period of VigiBase provide valuable real-world safety insights that complement randomized controlled trial data. The consistency of our findings with established pharmacological principles and prior clinical studies supports their validity as hypothesis-generating associations warranting further investigation through controlled studies.

Future research directions

Our findings highlight several important areas for future research. First, prospective clinical studies are needed to validate the pharmacodynamic correlations observed in this pharmacovigilance analysis. Randomized controlled trials comparing SSRIs with different receptor binding profiles could provide definitive evidence for mechanistic relationships between pharmacodynamics and safety outcomes. Second, the development of predictive models incorporating pharmacodynamic properties, pharmacokinetic parameters, and patient-specific factors could enhance precision prescribing approaches. Such models might incorporate genetic polymorphisms affecting drug metabolism, receptor expression, or neurotransmitter function to optimize treatment selection. Third, long-term observational studies are needed to assess the clinical significance of safety differences observed in pharmacovigilance databases. While our analysis demonstrates differential adverse event reporting patterns, the impact of these differences on treatment outcomes, quality of life, and healthcare utilization requires investigation through real-world effectiveness studies.

## Conclusions

This study provides the largest comparative real-world evaluation of the safety profiles of six widely used SSRIs. The findings demonstrate that SSRIs are not a uniform drug class but differ meaningfully in their adverse event patterns, largely reflecting their underlying pharmacodynamic properties. Paroxetine showed higher anticholinergic and withdrawal-related reporting, while citalopram exhibited stronger cardiac-related signals, emphasizing the need for drug-specific risk assessment. These results support a more individualized approach to SSRI prescribing to improve safety and tolerability. Future research should validate these observations and further refine precision-based antidepressant selection.

## Figures and Tables

**Figure 17:**
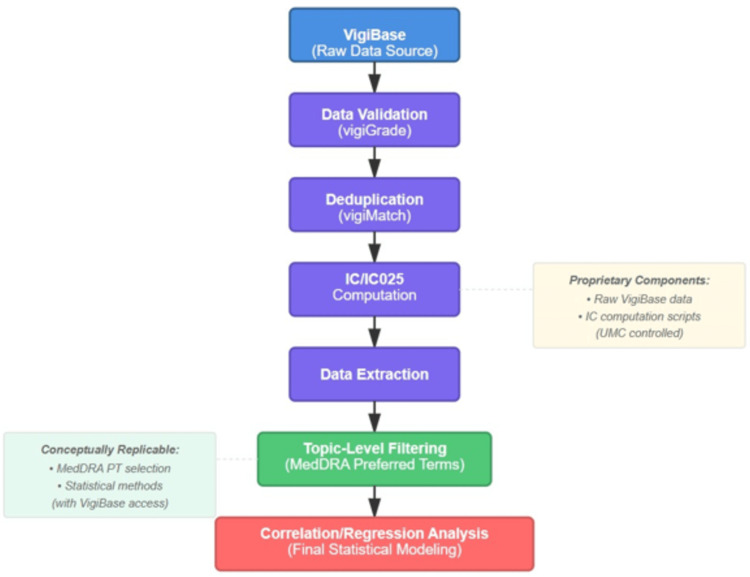
VigiBase data flow and analytical framework

## Appendices

**Table 4:** Summary of data processing steps in the VigiBase-based analysis

Step	Description	Responsible Entity	Reference
1	Submission of Individual Case Safety Reports (ICSRs) by national pharmacovigilance centres under WHO PIDM	WHO PIDM Member States	[19]
2	Data validation, anonymization, and upload to VigiBase	UMC	[19]
3	Coding of drugs (WHODrug Global) and adverse reactions (MedDRA v27.0)	UMC	[73]
4	Duplicate detection using vigiMatch algorithm	UMC	[74,75]
5	Dataset extraction (ER020-2024 snapshot, lock date: June 4, 2024)	UMC	Study Record
6	Signal detection via Information Component (IC, IC025) computation using Bayesian Confidence Propagation Neural Network (BCPNN)	UMC	[76,77]
7	Post-extraction correlation and regression analyses of IC values with receptor binding affinities and half-lives	Study Authors	Present Analysis
